# A novel internal fixation technique for chronic elbow dislocations: Technical note and case report

**DOI:** 10.1016/j.radcr.2025.05.059

**Published:** 2025-06-25

**Authors:** Amir Bisadi, Fatemeh Abbasi, Mohammad Abbasalizadeh, Morteza Gholipour

**Affiliations:** a Clinical Research Development Unit of Akhtar Hospital, Shahid Beheshti University of Medical Science, Tehran, Iran; b Student Research Committee, School of Medicine, Shahid Beheshti University of Medical Sciences, Tehran, Iran; c Student Research Committee, Faculty of Medicine, Mazandaran University of Medical Sciences, Mazandaran, Iran; d Student Research Committee, Tabriz University of Medical Sciences, Tabriz, Iran

**Keywords:** Neglected elbow dislocation, Surgical technique, Internal fixation, Case report, Technical note

## Abstract

Neglected elbow dislocations are difficult to treat and often lead to functional impairment, especially in resource-limited settings with limited surgical options. We report a case of a 40-year-old male who presented with a neglected elbow dislocation 8 weeks postinjury. Upon presentation, he exhibited a severely restricted range of motion and pain. The surgical approach involved the use of a modified T-plate adapted for resource-limited settings. Postoperatively, the elbow's range of motion was significantly enhanced, and stability was restored, demonstrating the efficacy of the adapted techniques.

## Introduction

Neglected chronic elbow dislocation, defined as nonreduced elbow dislocation for more than 3 weeks, presents a challenge in orthopedic practice, often resulting in significant functional impairment and decreased quality of life [1]. These dislocations are managed with open reduction, ligament repair, and often external fixation, but complications like nerve entrapment, heterotopic ossification, and joint misalignment are common [[1], [2], [3]]. In this study, we aimed to demonstrate the specific surgical technique employed to address the neglected elbow dislocation presented by the patient in a resource-limited setting.

## Case presentation

**Fig. 1:**
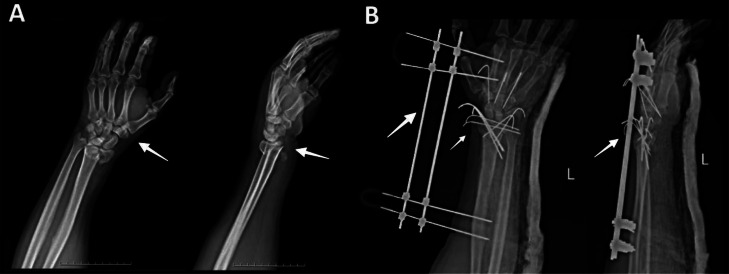
Radiographic assessment of distal radius fracture. (A) Anteroposterior (AP) and lateral X-ray views showing comminuted distal radius fracture (white arrows) with articular involvement and displacement. (B) Postoperative AP and lateral X-ray views demonstrating external fixator placement and K-wire fixation (white arrows) for management of the distal radius fracture and carpal-metacarpal injuries.

**Fig. 2:**
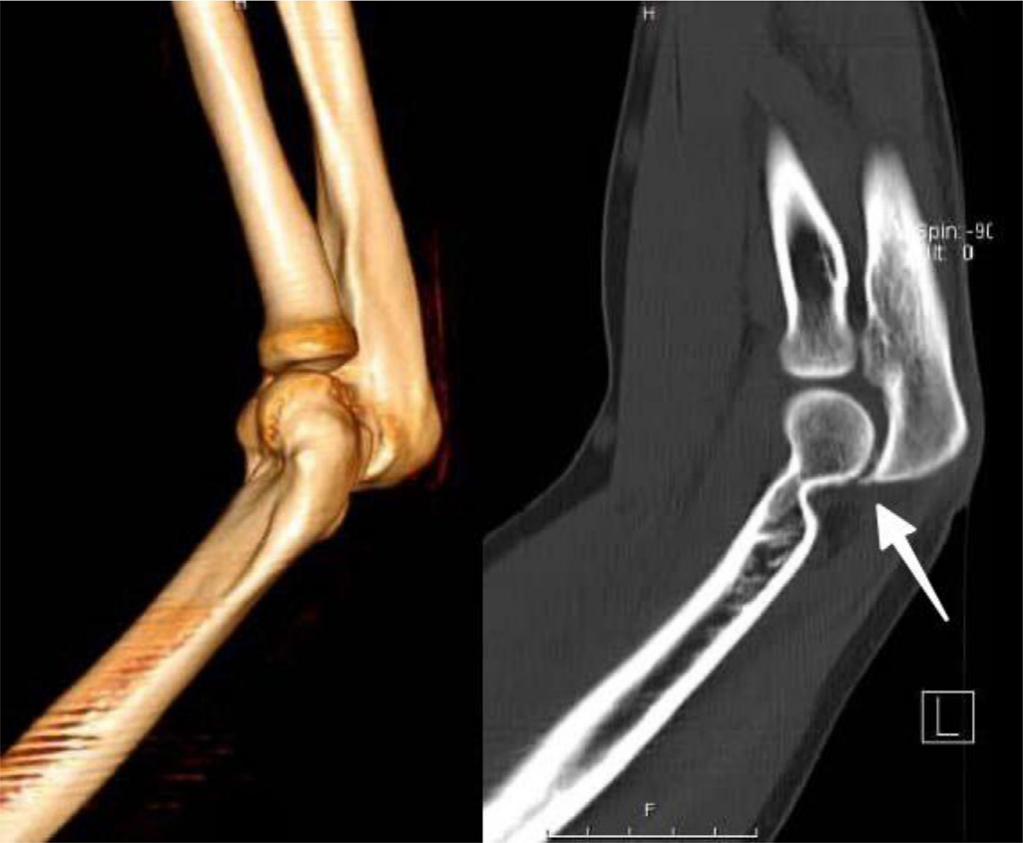
Preoperative CT imaging of the elbow joint. Left: 3D volume-rendered CT reconstruction in lateral view. Right: Sagittal CT reformation in bone window showing normal articulation between the olecranon and the trochlea (white arrow) without evidence of dislocation at initial presentation.

A 40-year-old male patient was delivered to the hospital after falling from a 3-meter height, sustaining trauma to the wrist and elbow. Wrist range of motion (ROM) was limited, and elbow ROM was painful. The neurovascular status of the affected hand was normal. Initial radiography revealed a compression-type distal radius fracture (AO/OTA class C3) and carpal metacarpal fractures, which were managed with closed reduction and percutaneous fixation using Kirschner Wire and an external fixator (Fig. 1). The elbow was not dislocated at the first presentation, as it is evident in the preoperative imaging (Fig. 2).

**Fig. 3:**
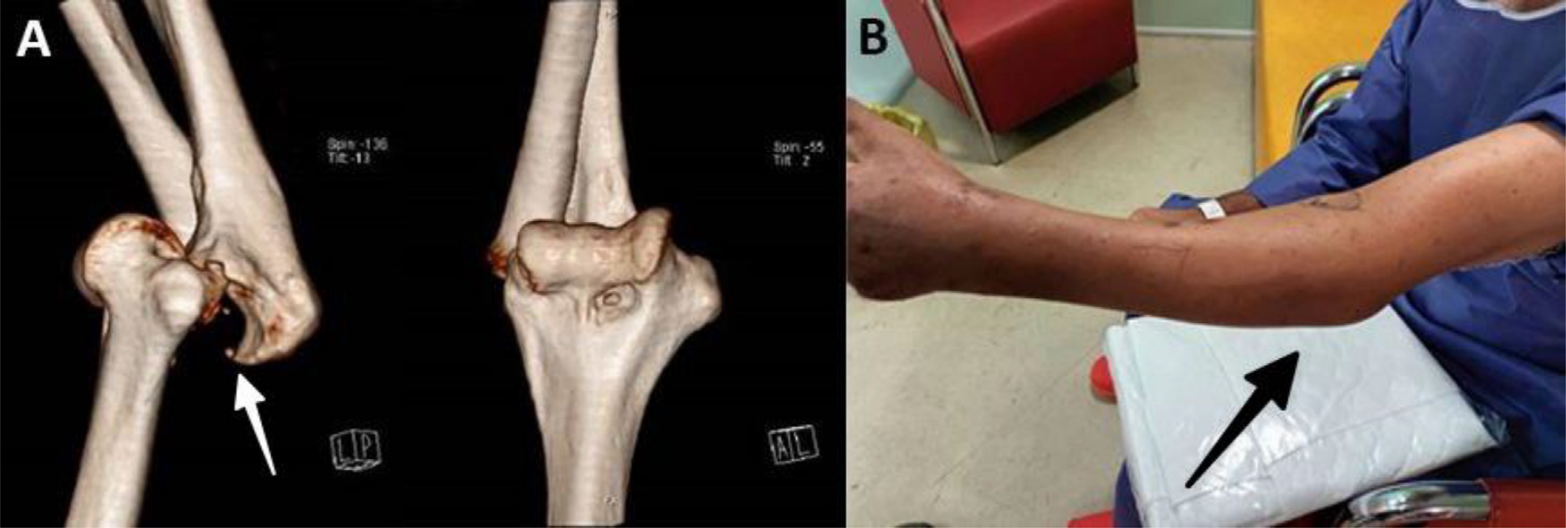
Documentation of neglected posterior elbow dislocation. (A) 3D volume-rendered CT reconstruction in lateral and AP views showing posterior displacement of the ulna relative to the distal humerus (white arrow) with loss of articulation between the trochlea and sigmoid notch. Note the disruption of the ulnohumeral line and dislocation of the radial head. (B) Clinical photograph demonstrating fixed flexion deformity and abnormal contour of the elbow joint (black arrow) with limited range of motion.

Six weeks after the surgery, the external fixator and pins were removed. The patient, who had not been cooperative regarding the course of follow-up, was presented to our center 8 weeks after the first surgery, at which point an elbow dislocation was identified upon examination. The patient had a flexion ROM of 40 degrees and an extension ROM of 20 degrees, not being able to perform supination and pronation (Fig. 3).

The patient was positioned supine on the operating table with the arm positioned on an arm board. An axial nerve block was performed. A pneumatic tourniquet was placed proximally on the arm and inflated to a pressure of 280 mmHg. The procedure began by identifying and preserving the ulnar nerve using a direct medial approach. An in situ neurolysis of the ulnar nerve was performed between the Osborne fascia and the arcade of Struthers. The ulnar collateral ligament (UCL) complex and the flexor mass were found to be completely torn and contracted. The wound was left open to observe the tension in the ulnar nerve during open reduction and distraction. Subsequently, Kocher’s interval was accessed through a separate lateral incision. The annular ligament was found to be torn and contracted. The anterior capsule was released, and fibrous tissue was removed. The lateral collateral ligament (LCL) was intact but was released subperiosteally, followed by lateral arthrolysis.

**Fig. 4:**
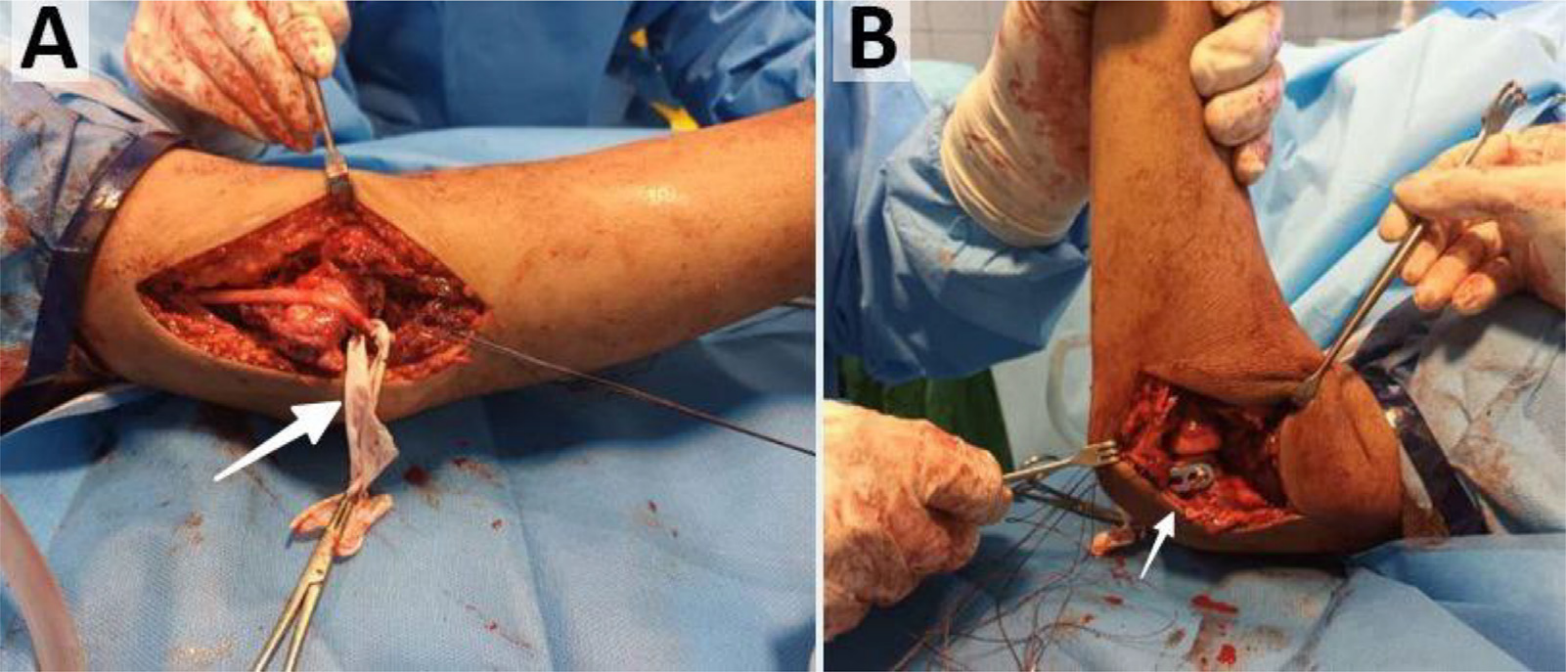
Intraoperative surgical management. (A) Intraoperative photograph showing medial approach with identification and anterior transposition of the ulnar nerve (white arrow) and capsular release. (B) Intraoperative photograph showing lateral approach with T-plate fixation (white arrow) spanning the reduced elbow joint.

An open reduction of the elbow joint was performed, and its stability was assessed. The annular ligament was repaired using the triceps fascia (Bell-Tawse procedure). The UCL and flexor muscles were repaired and secured with 5 mm suture anchors on the medial epicondyle of the humerus at their anatomical attachment in the medial epicondyle. The ulnar nerve was transpositioned anteriorly (Fig. 4).

**Fig. 5:**
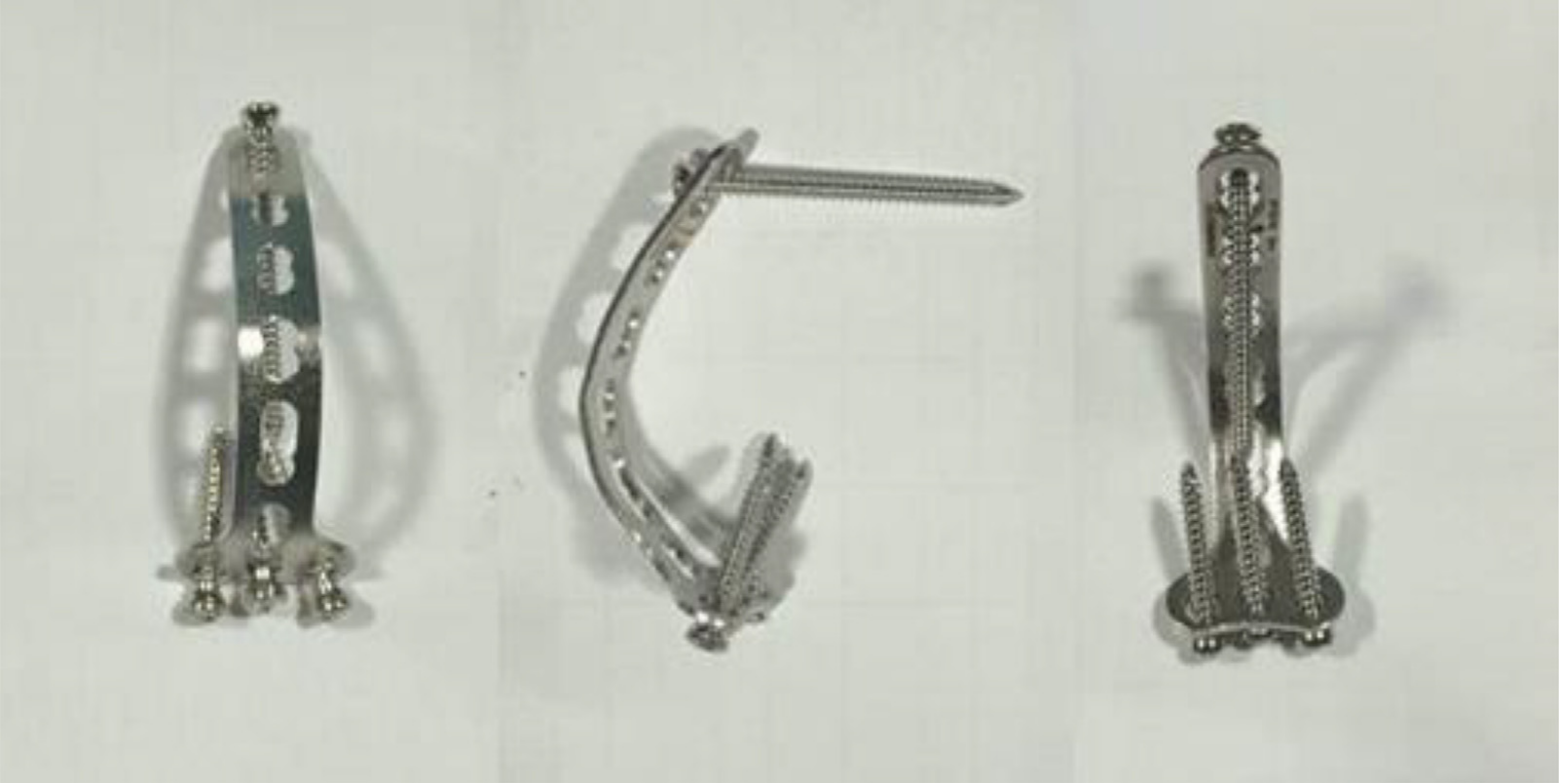
Modified T-plate design for elbow joint stabilization. Photograph of the curved T-plate from multiple angles showing the modified design with proximal segment for olecranon fixation and curved distal segment designed to allow for hinge movement at the center of rotation of the distal humerus.

To improve the stability of the elbow joint, internal fixation with a modified T-plate device was performed after ligament repair. A proximal section of the plate was placed at the proximal ulnar (Olecranon) and fixed using 3 cortical screws. The distal segment of the plate was curved and fixed at the center of rotation of the distal humerus and stabilized using a positional screw to allow for hinge movement of the elbow (Fig. 5).

The lateral collateral ligament and extensor muscles were then repaired and fixed using 5mm suture anchors at their anatomical site on the lateral humeral epicondyle. Then, the elbow was confirmed to be stable, and the ROM was assessed intraoperatively.

The patient’s arm was placed in a long arm splint for 1 week postoperatively and was allowed to be used at night for the next 3 weeks. Active ROM exercises for flexion and extension were initiated at the beginning of the first postoperative week. Physiotherapy commenced at the end of the second postoperative week.

**Fig. 6:**
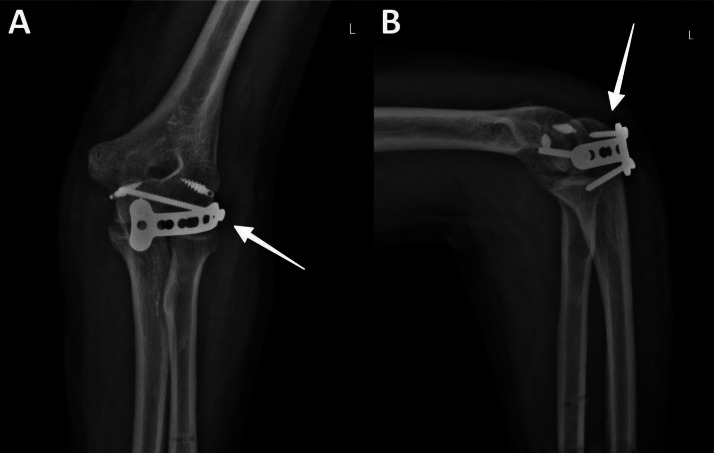
Postoperative radiographic assessment. (A) Anteroposterior X-ray view showing reduced elbow joint with T-plate fixation (white arrow) spanning the joint. The alignment of the ulnohumeral and radiohumeral joints is preserved. (B) Lateral X-ray view demonstrating congruent reduction of the ulnohumeral articulation with T-plate stabilization (white arrow) and appropriate screw placement in the olecranon and distal humerus. Note the restoration of the ulnohumeral line passing through the middle of the joint and the intersection of the radiocapitellar line with the capitellum, indicating proper reduction.

Postoperative radiographs are displayed in Fig. 6. Postoperative assessment on the 1-month follow-up demonstrated a flexion ROM of 100°, extension ROM of 10°, and nearly complete supination and pronation. The patient showed no signs of common complications such as wound infections, wound-related issues, or neurovascular complications.

Extended follow-up at 3-6 months postoperation reflected continued improvement in range, which was 110° flexion, 10° extension, and nearly complete supination and pronation (pronation 110°). Recurrence of dislocation was not seen. Prophylactic Indomethacin 25mg every 8 hours was given for 1 month postsurgery to prevent heterotopic ossification. Mild residual stiffness for terminal ranges for extension and pronation was noticed, but too minor to be a disability for activities. The patient, otherwise, was extremely pleased with the outcome and had satisfactory recovery for activities of daily living.

## Discussion

Here we report the management of a neglected elbow dislocation in a patient aged 40 with special reference to both the radiological features required to make the diagnosis and the unorthodox operating technique used. Clinical outcomes were optimal with good recovery of movement and pain relief.

Neglected elbow dislocations are associated with serious diagnostic and therapeutic challenges and cause widespread functional disability and a decrease in the quality of life [1]. They are also more prevalent in low-resource settings in which diagnosis and presentation are delayed and misdiagnosed [4,5].

## Radiological considerations in diagnosis

Our case presents the role of imaging in the underappreciated early and delayed diagnosis of elbow dislocation. While plain radiography is the first-line imaging modality of choice, our case demonstrates its shortcomings. As Rosas and Lee also note, conventional radiographs are subject to underestimation of joint relations in complex injuries [6].

CT scanning was definitive, both confirming the posterior dislocation and elucidating further information on concomitant bone injuries. The 3D reconstructions proved useful in preoperative management because spatial relationships of the joint surfaces were globally evaluable. This agrees with a report by Awad et al. [7], whereby 3-dimensional reconstruction by CT was shown to substantially boost diagnostic precision in complicated elbow trauma.

This elbow dislocation was radiologically imaged with emphasis on a number of key features, such as posterior displacement of the ulna, fractures of the coronoid process, if any, and heterotopic ossification. In our patient, prominent posterior displacement with minimal heterotopic ossification was found on the scans, which guided our surgical management.

While unattainable in our resource-constrained setup of circumstances, MRI has additional advantages in the evaluation of soft tissue injuries. Schreiber et al. elucidated MRI as a reliable indicator of collateral ligament injuries, capsular tears, and muscle contractures complicating reduction [8].

The radiological differentiation between acute and chronic dislocation is critical to surgery planning. Acute dislocation is radiographically revealed by signs of joint effusion, fat pad signs, swelling of the soft tissues, and hematoma, but has clean articular surfaces and scant adaptive changes. Chronic dislocation radiographically and on CT scan appears by periarticular fibrosis, heterotopic ossification, and remodeling of articular surfaces. We employed CT to determine the chronic changes and plan our surgery based on them. The 3-D reconstructions helped document best the degree of contracture, define the center of rotation as a prerequisite for best T-plate placement, and plan for serial releases. Although MRI would have been useful to assess the degree of soft tissue injury involving the ligamentous complex and leading to contractures, MRI was unavailable to us in our poverty-stricken environment. Management of future cases would be aided by MRI to further clarify the pathology within the soft tissues, and possibly change our plans appropriately.

## Surgical management considerations

Open reduction becomes unavoidable in neglected cases because of the formation of fibrotic tissue and contractures [2,3]. We used a combined medial and lateral approach with meticulous protection of the ulnar nerve, following the recommendations of Tuncali et al. [9], emphasizing neurolysis and anterior transposition of the ulnar nerve.

Our surgical technique required aggressive capsular release to address the soft tissue contractures that had developed during the 8-week period postinjury. Following reduction, we meticulously reconstructed the damaged ligamentous structures, including UCL repair and annular ligament reconstruction using the Bell-Tawse procedure, establishing a foundation for stability before applying our modified T-plate device.

The innovation in our technique was the utilization of a modified T-plate as an internal joint stabilizer. While standard procedures require the provision of conventional external fixation, the internal joint stabilizer (IJS) concept, as presented by Orbay et al., is intended to provide stability with early mobilization [10,11]. Our adaptation of the modified T-plate technique was planned to do the same thing in a resource-constrained setting.

We preferred a modified T-plate to a hinged external fixator for multiple reasons. First, hinged external fixators are difficult to obtain and expensive in our resource-poor environment, due to restrictions on importation. Second, there is poor patient acceptance for the use of an external fixator due to cosmetic considerations and functional restrictions. Third, after ligamentous repair, we had residual joint instability that required a stronger mode of stabilization. The modified T-plate gave us reliable stability and early motion, which is necessary to avoid heterotopic ossification, frequently seen after immobilization for long spans. This was better than the use of external fixation techniques, which was less reliable when it came to joint reduction, consistent, and less convenient when it came to removal of the implant. In operation, we used routine C-arm fluoroscopy as a replacement for dynamic fluoroscopy for determining satisfactory reduction on anteroposterior and lateral radiographs, for purposes of avoiding disruption of ligament repairs and increased radiation dosage.

Our approach to ligament reconstruction prioritized the key stabilizing structures of the elbow joint, focusing on restoring the integrity of the anterior bundle of the medial collateral ligament and the lateral collateral ligament complex. These critical structures were repaired using 5 mm suture anchors at their anatomical attachment sites on the respective humeral epicondyles to optimize joint stability. We prioritized their repair using suture anchors, and it was shown to be superior to direct repair alone by a study by McKee et al. [12].

Several fixation techniques are available for chronic elbow dislocation based on the chronicity and the condition of the joint (Table 1). Our method, modified T-plate, is a reasonable choice, keeping in view stability, early movement, and limited resources.

**Table 1:** Comparison of fixation methods for neglected elbow dislocations.

Fixation method	Advantages	Disadvantages	Recommended timeframe
Hinged external fixator	• Allows controlled motion • Maintains reduction while healing • Adjustable to accommodate soft tissue swelling • Can be removed without additional surgery	• Bulky apparatus • Pin tract infections • Patient discomfort • Higher cost in resource-limited settings • Lower patient acceptance	Typically used for dislocations >3 weeks
Modified internal joint stabilizer (Our T-plate method)	• Lower profile than external fixator • Allows early mobilization • Better patient acceptance • Provides stable fixation • More accessible in resource-limited settings	• Requires second surgery for removal • Potential for hardware failure • Less adjustability compared to external fixation	Effective for dislocations 3 weeks to 3 months postinjury
Olecranization	• Provides definitive treatment for severely damaged joints • Stable, predictable outcome • No concern for recurrent instability	• Sacrifices motion for stability • Permanent functional limitation • Technically demanding procedure	Reserved for dislocations >3 months with severe articular damage
Open reduction with ligament repair only	• Preserves natural joint mechanics • No additional hardware required • Simpler surgical technique	• May provide insufficient stability • Often requires prolonged immobilization • Higher risk of recurrent instability	Most effective for dislocations <3 weeks with minimal contracture

We initiated a prophylactic regimen of indomethacin (25 mg 8-hourly for 1 month) for heterotopic osteogenesis, a recognized complication of extensive operative manipulation for late elbow dislocation. This practice is based on published literature dealing with the use of prophylactic NSAIDs for the inhibition of heterotopic formation following elbow injury and surgery.

## Clinical outcomes and limitations

Our patient's functional recovery (ROM of flexion from 40° to 100° and extension from 10° to 20°) is consistent with outcomes cited in the literature. Mahaisavariya and Laupattarakasem documented an average flexion arc of 82.3° following conventional open reduction [13].

Our technique is supported by a study by Wynn et al. with similar clinical outcomes using internal joint stabilizers compared to external fixation, but fewer complications [14], and is a low-cost option and feasible in resource-poor settings.

As our case report is isolated, our results are not generalizable to others. Moreover, a single-month follow-up is too short and too early to decide long-term results. Long-term presentation of complications such as heterotopic ossification, recurrence of instability, and post-traumatic arthritis cannot be ruled out. Follow-up of a minimum of 12-24 months would have been necessary to evaluate durability and to screen for late complications.

Future studies must emphasize comparative analyses of various techniques of stabilization and standard radiological evaluation protocols. Other imaging techniques, like dynamic fluoroscopy or weight-bearing computed tomography (CT), might be able to offer significant information on functional joint stability after various procedures.

## Conclusion

This case presentation demonstrates the beneficial role of imaging diagnosis and management using novel surgical methods to address neglected elbow dislocations and the versatility of advanced methods in resource-poor settings. The interrelationship between prudent radiological assessment and accommodative surgical algorithms is a good model to treat such dismal injuries.

## Limitations

This report presents a case of neglected elbow dislocation treated by our modified technique using a T-plate device. Even though the result of this case is excellent, there are some important limitations. First, being an individual case report, our findings cannot apply to all neglected elbow dislocations. The success of our method must be confirmed by a series with a combination of groups of patients.

Even though we now have follow-up data for 3-6 months, which is favorable for functioning, the long-term follow-up is still too short for measurement overall. Later development of potential complications, such as heterotopic ossification, recurrent instability, post-traumatic arthritis, and loss of function, can be seen. A follow-up period of at least 12-24 months would be necessary to adequately determine durability, determine whether our outcome is durable over the long term, and detect late complications, which can only be seen after the passage of time.

More large-scale prospective studies with extended follow-up are needed to define the true efficacy, safety profile, and long-term outcome of such an altered procedure compared with conventional techniques.

## Data protection

All data utilized in this study were handled with confidentiality and anonymized. No identifying information about patients has been or will be revealed in the study findings or any subsequent publications.

## Ethical statement

This study was conducted in accordance with the principles outlined in the declaration of Helsinki. The research received approval from the Institutional Ethics Committee.

## Patient consent

All patients obtained informed consent and were informed about the study’s detail.
